# Unsupervised Approach Data Analysis Based on Fuzzy Possibilistic Clustering: Application to Medical Image MRI

**DOI:** 10.1155/2013/435497

**Published:** 2013-12-29

**Authors:** Nour-Eddine El Harchaoui, Mounir Ait Kerroum, Ahmed Hammouch, Mohamed Ouadou, Driss Aboutajdine

**Affiliations:** ^1^LRIT-CNRST URAC 29, Mohammed V-Agdal University, Faculty of Science, BP 1014, Rabat, Morocco; ^2^LARIT Equipe Imagerie et Multimedia, Ibn Tofail University, Faculty of Science, ENCG, BP 242, Kénitra, Morocco; ^3^LRGE, Mohammed V-Souissi University, ENSET, Rabat Instituts, BP 6207, Rabat, Morocco

## Abstract

The analysis and processing of large data are a challenge for researchers. Several approaches have been used to model these complex data, and they are based on some mathematical theories: fuzzy, probabilistic, possibilistic, and evidence theories. In this work, we propose a new unsupervised classification approach that combines the fuzzy and possibilistic theories; our purpose is to overcome the problems of uncertain data in complex systems. We used the membership function of fuzzy c-means (FCM) to initialize the parameters of possibilistic c-means (PCM), in order to solve the problem of coinciding clusters that are generated by PCM and also overcome the weakness of FCM to noise. To validate our approach, we used several validity indexes and we compared them with other conventional classification algorithms: fuzzy c-means, possibilistic c-means, and possibilistic fuzzy c-means. The experiments were realized on different synthetics data sets and real brain MR images.

## 1. Introduction

Image segmentation is a very important operation in the process of treatment and analyzing images, and it is widely used in the different fields: pattern recognition, remote sensing, artificial intelligence, medical imaging, and so forth. The field of medical imaging includes several types of images: radiography (X-ray), ultrasound and magnetic resonance image [1–4]. These images are a very complex data, so their analysis is a challenge for researches.

In the literature, there are several methods that can segment these images. We can group them in four classes. The first one is the *Thresholding*; it allows to find the optimal threshold value, in order to extract the background objects in the image. In general, this approach is very sensitive to noise and ignores the spatial parameters [5, 6].

The second approach is the *Contour*; it allows to detect the contour of the image. This method is easy to implement, but unfortunately it is very sensitive to the noise and also to the parameters initialization, which means that it is mostly used with a pretreatment filter [7–10].

The third approach is the *Region*, which generates some methods: growing region (called ascendant) and splitting/merging (called descendants); this approach is very sensitive to the initial parameters and to the noise [11–13].

The last approach is the *Clustering*; it is a very important operation in the process and data analysis, and it allows creating the homogeneous partitions using a similarity criterion [3, 4, 14–33].

In this work, we are interested in clustering segmentation using the possibility theory combined with fuzzy theory.

The remainder of this paper is structured as follows. In Section 2, we present the clustering approach with three conventional algorithms, fuzzy c-means (FCM) (Algorithm 1), possibilistic c-means (PCM) (Algorithm 2), and possibilistic fuzzy c-means (PFCM) (Algorithm 3). In Section 3, the proposed new approach (HPFCM) is formulated to cluster the complex data set. In Section 4, we present the experimental results using different synthetics data sets and real MR images. Finally, the conclusion and perspectives are presented in Section 5.

## 2. Theories of Data Analysis

### 2.1. Classification Approach

Classification is a method that allows analysing and treating the complex data. It is widely used in different fields: pattern recognition, remote sensing, images processing, and artificial intelligence [3, 14, 16].

In the literature there are two types of classification: supervised and unsupervised. In general, the supervised approach uses a learning base in order to extract and produce the decision functions for classifying data. But, the unsupervised classification is used without a learning knowledge base, also called clustering [17, 22, 34, 35].

In this work, we are interested in studying clustering approach and its applications in medical image processing.

### 2.2. Clustering

Clustering is a process that allows dividing data into groups of similar pattern, and these groups are called clusters. In the literature, there are several theories of clustering. The first notion of fuzzy theory was proposed by Zadeh [36], and he established the basic principle of fuzzy theory using the fuzzy logic, in order to describe the uncertainty of belonging by a membership function. Then Ruspini [35] proposed the first notion of a fuzzy partition, and he considers that each cluster is a fuzzy set. Zadeh [34] proposed the conceptual framework for cluster analysis and pattern classification using the fuzzy set theory. Later, several researches were published; in order to improve the Bezdek algorithm, Rousseeuw et al. [37–39] proposed the different objective functions that allow improving the effectiveness of fuzzy c-means algorithm.

To overcome the weakness of FCM to noise, Krishnapuram and Keller [23] proposed to relax the constraint of fuzzy, and they established the first possibilistic c-means algorithm (PCM). Then Barni et al. [40] showed that PCM algorithm is very sensitive to initializations and generates the coincident clusters. Timm et al. [29] proposed the possibilistic fuzzy clustering, and Pal et al. [30] proposed another possibilistic fuzzy c-means (PFCM) that can avoid the coincident cluster of PCM and be less sensitive to the noise.

### 2.3. Fuzzy c-Means

Fuzzy c-means algorithm was established by Bezdek [22], it allows classifying uncertain and imprecise data, and it is the most widely used in fuzzy clustering. The FCM model is the optimization problem *J*_*m*_:

(1) $$\begin{array}{c} J_{m}\left(U,V;X\right)=\sum_{i=1}^{N}\sum_{j=1}^{K}u_{ij}^{m}{||x_{i}-v_{j}||}_{A}^{2}, \end{array}$$

where *X* = {*X*_*i*_, *i* = 1 ⋯ *N*}⊆*ℜ* ^*q*^ is the data set, *n* is the number of data, *K* is the number of clusters, *m* is the degree of fuzzy, *u*_*ij*_ is the degree of membership, *v*_*j*_ is the center of cluster *j*, and ||*x*_*i*_ − *v*_*j*_||_*A*_ is a distance between *V*_*j*_ and the object *x*_*i*_. Consider

(2) ΨKN={U∈ℜNK:0≤uij≤1,  ∀i,∀j;  ∀i∃j  uij>0},

(3) Mfc={U∈ΨKN:∑j=1Kuij=1,  ∀i∈{1,…,N};  ∑i=1Kuij>0,  ∀j∈{1,…,N}}.

**Theorem 1 (FCM)**

If *D*_*ijA*_ = ||*x*_*i*_−*v*_*j*_||_*A*_ > 0, for all *i*, *j*, *m* > 1, and *X* data set contains at least *K* different patterns, then (*U*, *V*) ∈ *M*_*fc*_ × *ℜ* ^*K*×*q*^ and *J*_*m*_ can be minimized only if

(4) uij=(∑s=1K(||xi−vj||A2||xs−vj||A2)1/(m−1))−1,   i∈{1,…,N},j∈{1,…,K},

(5) $$\begin{array}{c} v_{j}=\frac{\sum_{i=1}^{N}u_{ij}^{m}x_{i}}{\sum_{i=1}^{N}u_{ij}^{m}},\;\forall j\in \left\{1,\ldots ,K\right\}. \end{array}$$

### 2.4. Possibilistic c-Means

Possibilistic c-means algorithm was introduced by Krishnapuram and Keller [23] to overcome the sensitivity of fuzzy c-means to noise. Their idea allows relaxing the fuzzy constraint.

The PCM is the optimization problem *J*_pcm_:

(6) Jpcm(U,V;X,η) ={∑i=1N∑j=1Kuijm||xi−vj||A2+∑j=1Kηj∑j=1N(1−uij)m},0≤uij≤1,  ∀i∈{1,…,N}, ∀j∈{1,…,K}.

**Theorem 2 (PCM)**

If *D*_*ijA*_ = ||*x*_*i*_−*v*_*j*_||_*A*_ > 0, for all *i*, *j*, *m* > 1, *η* > 1, and *X* data set contains at least *K* different patterns, then (*U*, *V*) ∈ Ψ_*KN*_ × *ℜ* ^*K*×*q*^ and *J*_*pcm*_ can be minimized only if

(7) uij=((1+(||xi−vj||A2ηj)))−1/(m−1),       1≤i≤N,  1≤j≤K,

(8) $$\begin{array}{c} v_{j}=\frac{\sum_{i=1}^{N}u_{ij}^{m}x_{i}}{\sum_{i=1}^{N}u_{ij}^{m}},\;\forall j\in \left\{1,\ldots ,K\right\}. \end{array}$$

### 2.5. Possibilistic Fuzzy c-Means (PFCM)

Pal et al. [30] proposed the possibilistic fuzzy c-means algorithm (PFCM) that is based on FCM and PCM, and it generates the two membership functions concurrently; the first is a possibilistic membership (*t*_*ij*_) that presents the absolute degree of typicality, and the second function is the fuzzy membership (*u*_*ij*_) that presents the relative degree.

The PFCM is the optimization problem *J*_pfcm_:

(9) Jpfcm(U,V;X,η) ={∑i=1N∑j=1K(auijm+btijη)||xi−vj||A2+∑j=1Kγj∑j=1N(1−tij)η},

where *a* > 0, *b* > 0, *m* > 1, and *η* > 1.

**Theorem 3 (PFCM)**

If *D*_*ijA*_ = ||*x*_*i*_−*v*_*j*_||_*A*_ > 0, for all *i*, *j*, *m* > 1, *η* > 1, and *X* data set contains at least *K* different patterns, then (*U*, *T*, *V*) ∈ *M*_*fc*_ × Ψ_*KN*_ × *ℜ* ^*K*×*q*^ and *J*_*pfcm*_ can be minimized only if

(10) uij=(∑s=1K(||xi−vj||A2||xs−vj||A2)1/(m−1))−1,       1≤i≤N,  1≤j≤K,

(11) tij=11+((b/γj)||xi−vj||A2)1/(η−1),      1≤i≤N,  1≤j≤K,

(12) $$\begin{array}{c} v_{j}=\frac{\sum_{i=1}^{N}\left(au_{ij}^{m}+bt_{ij}^{\eta }\right)x_{i}}{\sum_{i=1}^{N}\left(au_{ij}^{m}+bt_{ij}^{\eta }\right)},\;1\leq j\leq K. \end{array}$$

Pal et al. [30] showed that PFCM can overcome the weakness of FCM to noise and also overcome the problem of coincident class of PCM.

## 3. The Proposed Algorithm HPFCM

### 3.1. HPFCM

Our new hybrid approach HPFCM is based on the previous two mathematical theories, the theory of fuzzy sets and possibility theory. Our contribution to hybrid HPFCM uses the results of the FCM algorithm as input data for the PCM algorithm. The degree of membership matrix PCM is initialized by the value of the result matrix of FCM. We present our algorithm in Algorithm 4.

## 4. Experimental Results 

### 4.1. Synthetic Data Sets

*Data Set X*_12_. This data set consists of 10 patterns and two outliers (noise), as shown in Table 1 and as given in [30]. We applied the FCM, PCM, PFCM, and HPFCM to *X*_12_ with initial parameters *m* = 2, *K* = 2 (number of clusters), and *η* = 2 and the center matrix $V\left[\begin{array}{cc} v_{1} & v_{2} \end{array}\right]$ with random values. The ideal (true) centroids are

(13) $$\begin{array}{c} V_{\text{ideal}}=\left[\begin{array}{cc} -3.14 & 0\\ 3.14 & 0 \end{array}\right]. \end{array}$$

Table 2 shows the results of prototypes of center clusters *v*_1_ and *v*_2_ using the four algorithms. For calculating the error between the results prototypes and ideal center clusters, we used the formula: Er = ||*V*_ideal_−*V*_∗_||^2^, where ∗ is the FCM, PCM, PFCM, and HPFCM. So, we can have the errors as follows: Er_fcm_ = 0.414, Er_pcm_ = 1.416, Er_pfcm_ = 0.3796, and Er_hpfcm_ = 0.0488.

**Figure 1:**
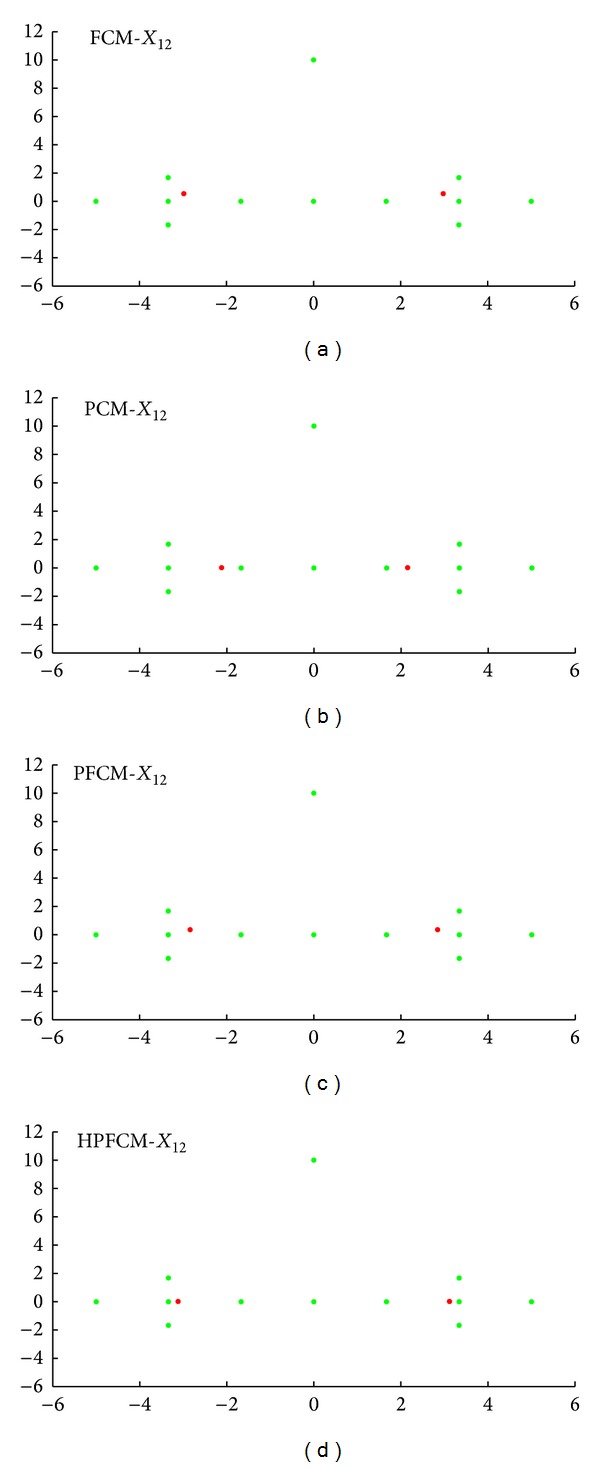
Clustering results of four algorithms FCM, PCM, PFCM, and HPFCM with data set *X*_12_.

These error values show that our method gives the best prototypes for *v*_1_ and *v*_2_. And Figure 1 shows the effectiveness of our approach.

### 4.2. Real Data Sets of UCI 

#### 4.2.1. Description of UCI Data Sets

In this section, we tested the performance of our method in different real data sets: *Iris*, Glass, Wine, and Wisconsin Breast cancer, as shown in Table 3. These data sets are referenced in Benchmark of UCI Repository of Machine Learning Data base [31].

*Iris Data Set.* It describes a type of *Iris* plant, and it contains 3 classes of 50 instances for each class. There is one class (class 1) which is linearly separable from the other, but the classes 2 and 3 are not linearly separable, and it is a four-dimensional data set [31].

*Wine Data Set.* The Wine is a 13-dimensional data set and contains the values of a chemical analysis of Wines grown in the same region in Italy but derived from three different cultivars [31].

*Glass Data Set.* Glass is a 9-dimensional data set that contains 214 objects of 6 classes representing the types of glass [31].

*Breast Cancer Data Set.* The Wisconsin Breast cancer is a 9-dimensional data set that contains 699 objects of 2 classes representing the types of benign and malignant cancers [31].

#### 4.2.2. Clustering Results for UCI Data Sets

To evaluate the clustering accuracy, we used the index of Huang and Ng [32] as given in

(14) $$\begin{array}{c} h=\frac{\sum_{i=1}^{K}c_{i}}{n}, \end{array}$$

where *K* is the number of clusters, *c*_*i*_ is the number of patterns occurring in both cluster *i*th and its true corresponding, and *n* is the total number of objects in the data set.

In Table 4, using *Iris* data set, we can see that PCM algorithm gave less accuracy (0.667) than other algorithms, and the algorithms FCM and PFCM gave the values 0.893 and 0.9, respectively, and our approach gave the best accuracy (0.929). And using other data sets, Wine, Glass, and Breast, we can also see that all accuracy values of our approach are better compared to other algorithms: FCM, PCM, and PFCM.

### 4.3. Medical MR Images Data Sets 

#### 4.3.1. Validity Index

**Figure 2:**
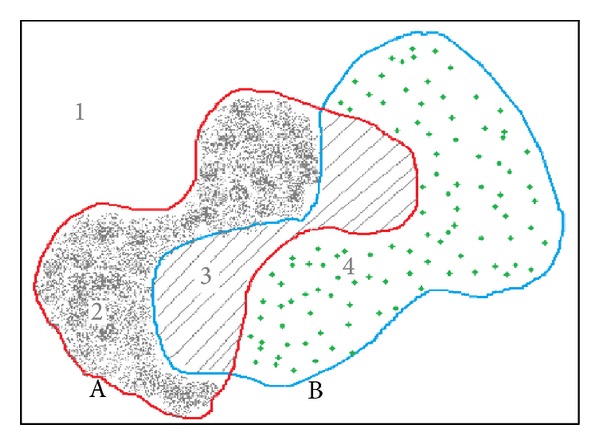
Description of the sets.

There are several validity indexes to evaluate the performance of a clustering method [25]. In our work, we used the sensitivity index (Se), specificity (Sp), and the classification accuracy (AC). These three indexes are based on the four parameters: true positive (TP), true negative (TN), false positive (FP), and false negative (FN), shown in Figure 2, Tables 5 and 6.

- TP is a true positive, when a pixel labeled is correctly classified as positive.
- TN is a true negative, when a pixel labeled is correctly classified as negative.
- FP is a false positive, when a negative labeled pixel is incorrectly classified as positive.
- FN is a false negative, when a positive labeled pixel is incorrectly classified as negative.

Sensitivity (Se) index gives the proportion of true positives over all structures that should be segmented

(15) $$\begin{array}{c} \text{Se}=\frac{\text{TP}}{\text{TP}+\text{FN}}. \end{array}$$

Specificity (Sp) index gives the proportion of true negatives over all structures, that should not be segmented

(16) $$\begin{array}{c} \text{Sp}=\frac{\text{TN}}{\text{TN}+\text{FP}}. \end{array}$$

Classification accuracy (CA) index gives the performance of a method:

(17) $$\begin{array}{c} \text{CA}=\frac{\text{TP}+\text{TN}}{\text{TP}+\text{FN}+\text{TN}+\text{FP}}. \end{array}$$

#### 4.3.2. Brainweb: Simulated Brain Database

**Figure 3:**
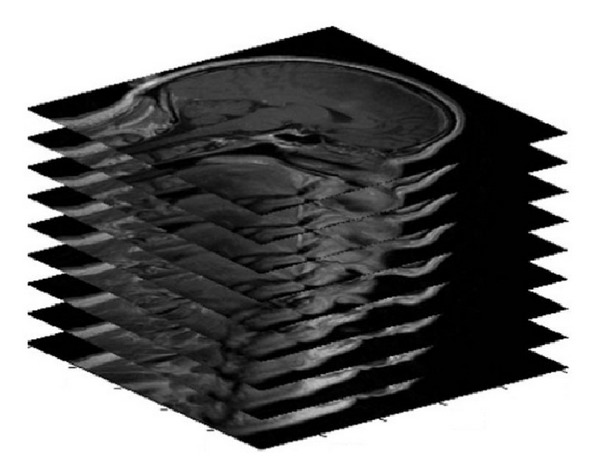
Brain MR image 3D.

**Figure 4:**
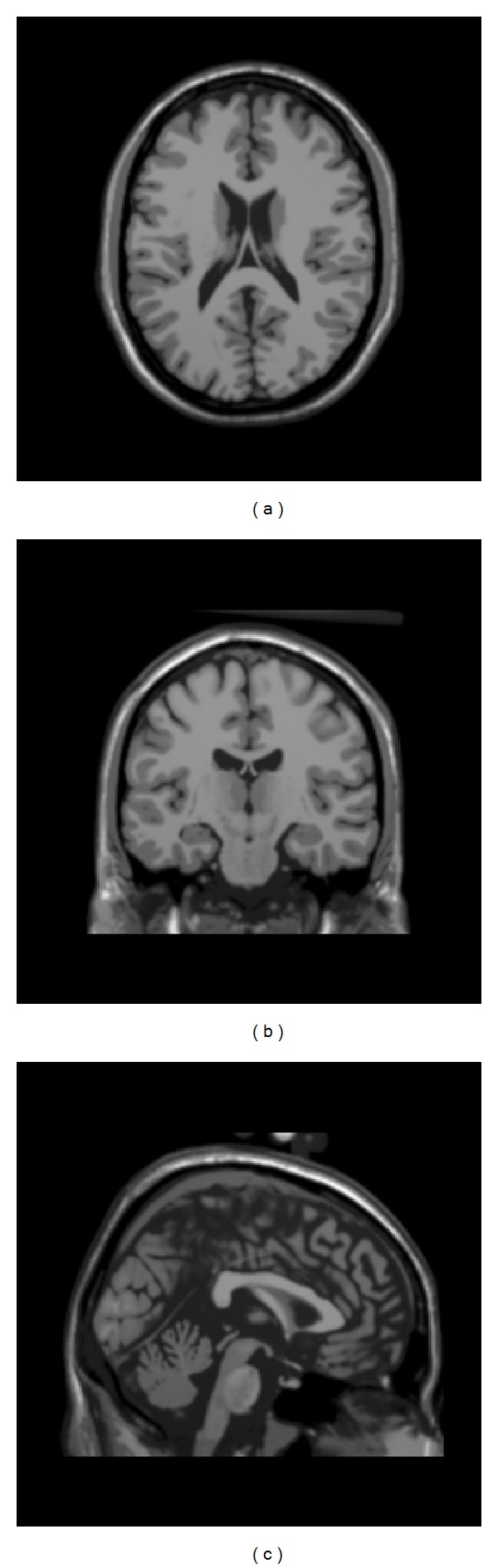
Brain MR images with three views axial, coronal, and sagittal [41].

**Figure 5:**
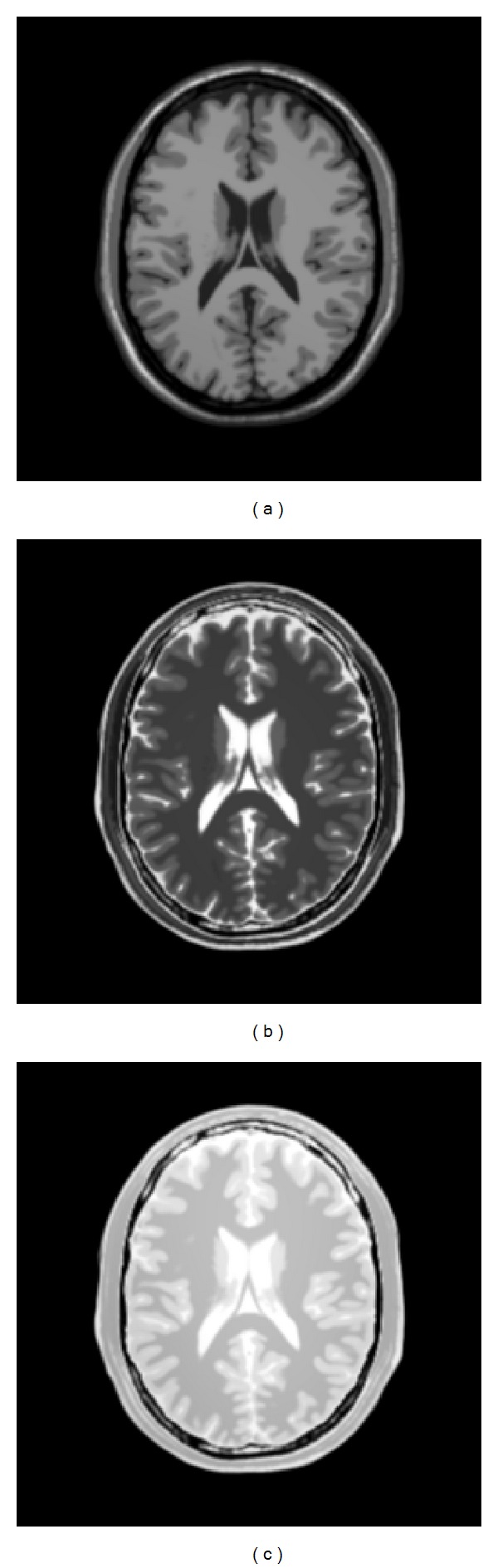
Brain MR images with three sequences volumes T1, T2, and DP [41].

This data set can simulate the brain MR images in three orthogonal views (transversal, sagittal, and coronal); it can also give three sequences volumes (T1, T2, and PD (proton density)), as shown in Figures 3, 4, and 5 [41], and a variety of slice thicknesses, noise levels, and levels of intensity nonuniformity [41].

**Figure 6:**
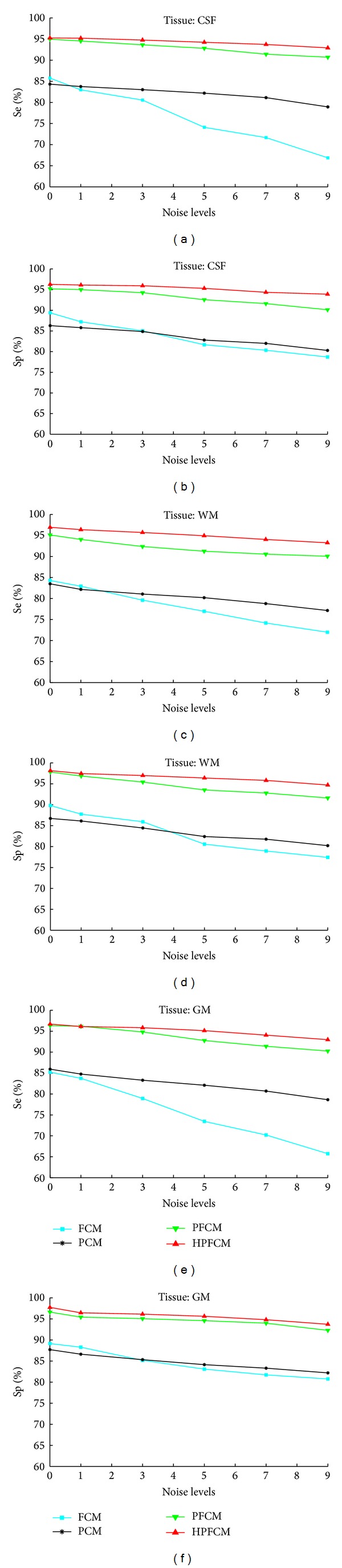
Curves of clustering index (Se and Sp) for CSF, WM, and GM, using four algorithms, FCM, PCM, PFCM, and HPFCM.

**Figure 7:**
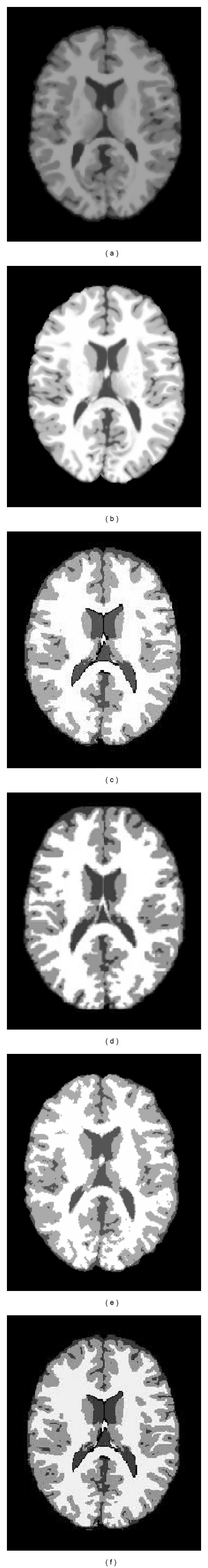
Clustering results using four algorithms: (a) original image, (b) Ground Truth, (c) FCM, (d) PCM, (e) PFCM, and (f) HPFCM, using brain MR images from Brainweb.

Tables 7, 8, and 9 show the clustering results using the different brain tissues: gray matter (GM), white matter (WM), and cerebrospinal fluid (CSF), respectively. We used the different noise levels (0%, 1%, 3%, 5%, 7%, and 9%), in order to evaluate our approach HPFCM and we compared the accuracy results with other algorithms, FCM, PCM, and PFCM. We can see that FCM algorithm is not stable and very sensitive to noises, and PCM algorithm gave stable values, but it did not show enough performance; instead PFCM and HPFCM gave good performance and were stable. Also, we plotted the curves for visualizing the results, as can be seen in Figure 6, that our approach gave the best accuracy compared to PFCM and other conventional algorithms FCM and PCM. The clustering results for brain MR image with Ground Truth are shown in Figure 7.

#### 4.3.3. IBSR Data Set

To evaluate our approach on real medical images, we used the IBSR data sets (Internet Brain Segmentation Repository), which is given by the Center for Morphometric Analysis of Massachusetts General Hospital [42].

Table 10 shows the clustering results of different brain tissues: GM, WM, and CSF. We did the study on 10 images from data sets IBSR; each image has 150 × 256 × 256 voxels. Also, the Ground Truth was established by the experts and it is available in IBSR [42].

**Figure 8:**
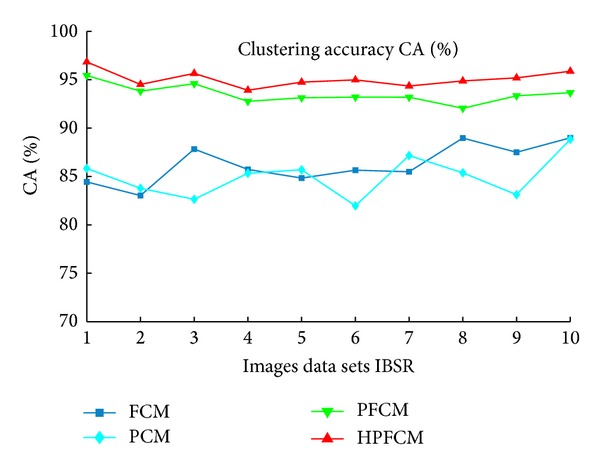
Results of clustering accuracy for the algorithms, FCM, PCM, PFCM, and HPFCM, using MR images from IBSR.

**Figure 9:**
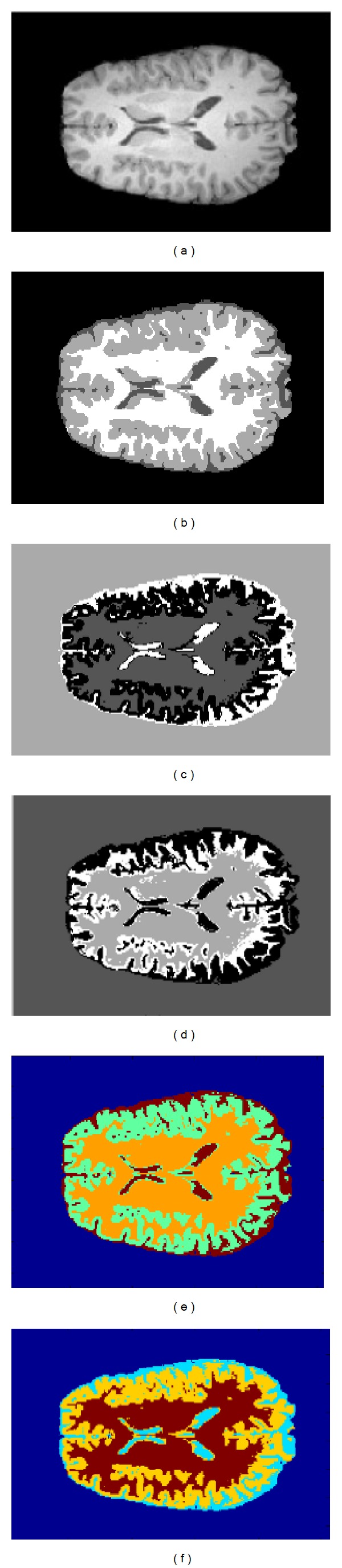
Clustering results using four algorithms: (a) original image, (b) Ground Truth, (c) FCM, (d) PCM, (e) PFCM, and (f) HPFCM, using brain MR images from IBSR data sets.

According to the results of Table 11, we can see that our approach HPFCM gave the best results using different indexes: specificity (Se), sensitivity (Sp), and clustering accuracy (CA). In order to better analyze the results, we established the curve, as shown in Figure 8, so this curve shows that our approach is a better model and very efficient compared to other algorithms FCM, PCM, and PFCM. The clustering results of brain MR images of IBSR with Ground Truth are shown in Figure 9.

## 5. Conclusion

In this paper, we presented a new approach to clustering, which is based on the fuzzy c-means and possibilistic c-means. Our approach is used to model uncertain and imprecise data, in order to segment the brain tissue for medical images MRI. And it has been compared to three conventional clustering algorithms: FCM, PCM, and PFCM. The performance of our approach has been successfully validated on several synthetics data sets and real medical images MRI. In the future, we will integrate other theories: the genetic and evidence theory, in order to optimize our hybrid method and we can have a very robust modeling for complex data.

## Figures and Tables

**Algorithm 1:**
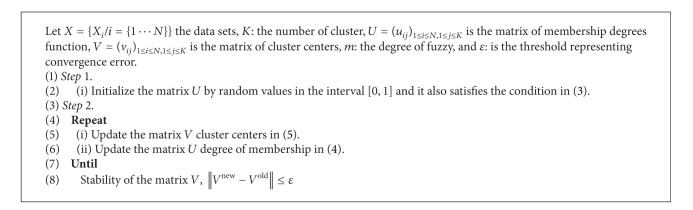
FCM.

**Algorithm 2:**
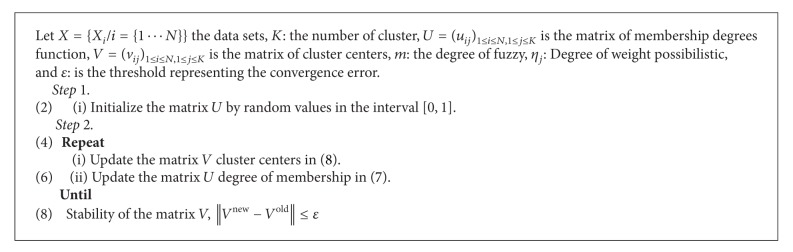
PCM.

**Algorithm 3:**
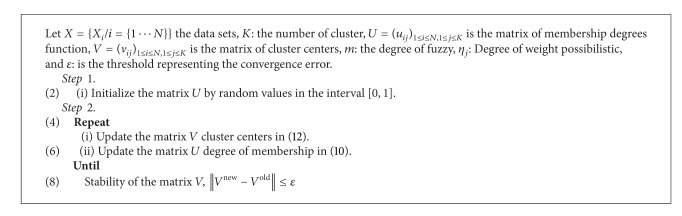
PFCM.

**Algorithm 4:**
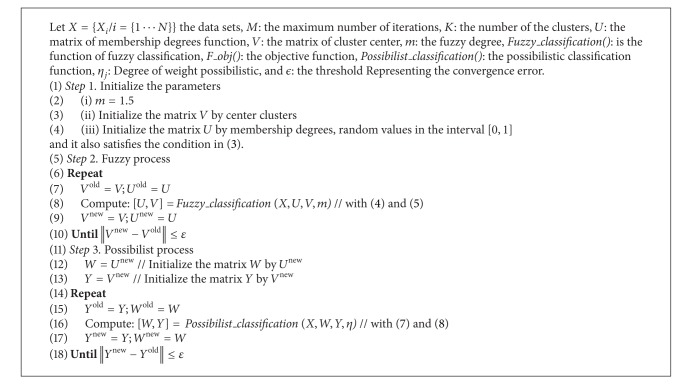
HPFCM.

**Table 1:** Data sets *X*_12_.

	*X*	*Y*
1	−5.00	0.00
2	−3.34	1.67
3	−3.34	0.00
4	−3.34	−1.67
5	−1.67	0.00
6	1.67	0.00
7	3.34	1.67
8	3.34	0.00
9	3.34	−1.67
10	5.00	0.00
11	0.00	0.00
12	0.00	10.00

**Table 2:** Prototypes results of *V*, produced by FCM, PCM, PFCM, and HPFCM with data set *X*_12_.

FCM		PCM		PFCM		HPFCM	
(*m* = 2, *η* = 2)		(*η* = 2)		(*a* = 1, *b* = 1, *m* = 2, *η* = 2)		(*m* = 2, *η* = 2)	
*υ*_1_	*υ*_2_	*υ*_1_	*υ*_2_	*υ*_1_	*υ*_2_	*υ*_1_	*υ*_2_
−2.98	2.98	−2.15	2.15	−2.84	2.84	−3.12	3.12
0.54	0.54	0.02	0.02	0.36	0.36	0.02	0.02

**Table 3:** Characteristics of data sets: *Iris*, Wine, Breast-w, and Glass. Ref. data sets UCI [31].

Data sets	No. objet	No. attribute	No. class	No. each cluster
*Iris *	150	4	3	50,50,50
Wine	178	13	3	48,59,71
Breast-w	699	9	2	241,458
Glass	214	9	6	9,29,13,70,17,76

**Table 4:** Clustering accuracy by index Huang for FCM, PCM, PFCM, and HPFCM with four data sets: *Iris*, Wine, Glass, and Breast cancer.

Data sets	FCM	PCM	PFCM	HPFCM
*Iris *	0.893	0.667	0.900	0.929
Wine	0.685	0.415	0.700	0.789
Breast-w	0.843	0.618	0.863	0.891
Glass	0.721	0.554	0.823	0.876

**Table 5:** Index description.

	Truth		
		Yes	No
Classification	Yes	Region-3(TP)	Region-2(FP)
	No	Region-4(FN)	Region-1(TN)

**Table 6:** Regions description.

	Description	Index
Region 1	(∁_*A*_∩∁_*B*_)	TN
Region 2	(*A*∖(*A*∩*B*))	FP
Region 3	(*A*∩*B*)	TP
Region 4	(*B*∖(*A*∩*B*))	FN

∁_*A*_	Complement of region (*A*)	
∁_*B*_	Complement of region (*B*)	
∩	Operation of intersection	
*∖*	Operation of difference	

**Table 7:** Results comparison for clustering index (Se and Sp) for GM tissue by FCM, PCM, PFCM, and HPFCM, with different noises (0%, 1%, 3%, 5%, 7%, and 9%), using Brainweb data sets.

Noise	FCM		PCM		PFCM		HPFCM	
	Se	Sp	Se	Sp	Se	Sp	Se	Sp
0%	85.19	89.15	85.93	87.71	96.33	96.61	96.69	97.73
1%	83.73	88.28	84.75	86.63	96.18	95.42	96.11	96.45
3%	78.92	85.17	83.28	85.32	94.81	95.05	95.82	96.13
5%	73.45	83.09	82.09	84.14	92.77	94.58	95.13	95.62
7%	70.20	81.72	80.69	83.31	91.39	93.98	94.05	94.81
9%	65.73	80.75	78.63	82.19	90.26	92.28	92.97	93.72

**Table 8:** Results comparison for clustering index (Se and Sp) for WM tissue by FCM, PCM, PFCM, and HPFCM with different noises (0%, 1%, 3%, 5%, 7%, and 9%), using Brainweb data sets.

Noise	FCM		PCM		PFCM		HPFCM	
	Se	Sp	Se	Sp	Se	Sp	Se	Sp
0%	83.30	89.81	83.50	86.72	95.12	97.82	96.95	98.14
1%	82.92	87.14	82.17	86.11	94.06	96.85	96.38	97.45
3%	79.61	85.92	81.06	84.44	92.37	95.41	95.71	96.97
5%	76.96	80.57	80.20	82.39	91.25	93.52	94.94	96.38
7%	74.18	78.93	78.79	81.76	90.55	92.79	94.05	95.81
9%	71.99	77.41	77.15	80.22	90.06	91.60	93.26	94.70

**Table 9:** Results comparison for clustering index (Se and Sp) for CSF tissue by FCM, PCM, PFCM, and HPFCM, with different noises (0%, 1%, 3%, 5%, 7%, and 9%), using Brainweb data sets.

Noise	FCM		PCM		PFCM		HPFCM	
	Se	Sp	Se	Sp	Se	Sp	Se	Sp
0%	85.77	89.39	84.32	86.28	94.98	95.19	95.27	96.28
1%	82.98	87.22	83.76	85.79	94.54	95.00	95.21	96.10
3%	80.55	85.02	83.02	84.86	93.63	94.26	94.77	95.93
5%	74.10	81.66	82.19	82.79	92.81	92.56	94.24	95.31
7%	71.66	80.32	81.63	81.99	91.42	91.61	93.72	94.35
9%	66.87	78.69	78.94	80.28	90.70	90.13	92.91	93.89

**Table 10:** Clustering by FCM, PCM, PFCM, and HPFCM, using IBSR dataset.

	Images	FCM		PCM		PFCM		HPFCM	
		Se	Sp	Se	Sp	Se	Sp	Se	Sp
GM	IM1	78.13	88.89	82.36	86.77	90.34	96.32	91.67	97.13
	IM2	81.87	85.05	87.56	87.17	91.20	95.25	93.78	96.43
	IM3	86.75	87.84	84.23	83.32	95.75	95.63	97.34	96.87
	IM4	85.56	85.74	85.76	86.76	91.23	94.21	90.89	94.54
	IM5	88.11	88.53	85.43	83.98	93.47	94.73	94.69	96.33
	IM6	85.65	87.97	82.47	84.31	93.88	95.98	96.45	96.72
	IM7	88.94	89.28	85.78	86.87	93.62	93.19	94.79	93.23
	IM8	86.44	86.59	87.07	85.65	91.07	95.23	90.23	96.55
	IM9	83.36	88.54	81.82	84.16	93.72	94.76	95.62	96.64
	IM10	86.67	89.93	87.39	86.66	94.66	95.55	96.80	97.75

WM	IM1	83.53	81.15	88.38	81.63	91.86	93.46	93.75	95.37
	IM2	82.96	83.85	83.33	84.44	95.20	91.17	96.06	91.83
	IM3	78.28	88.39	77.95	79.93	93.67	93.84	94.19	95.07
	IM4	85.4	82.19	84.96	82.06	91.91	92.62	92.84	92.13
	IM5	83.17	79.04	87.49	81.77	90.10	95.32	91.29	96.73
	IM6	82.51	81.27	79.86	78.21	93.33	91.07	95.39	91.99
	IM7	86.85	85.58	78.2	82.99	90.00	91.86	91.55	92.38
	IM8	78.55	89.61	83.11	88.29	91.85	90.27	93.43	92.59
	IM9	84.3	83.66	86.82	80.33	96.33	90.82	97.22	91.67
	IM10	80.24	82.85	81.07	81.08	90.21	91.55	90.87	93.32

CSF	IM1	74.75	79.85	82.49	84.95	91.11	92.83	93.75	94.75
	IM2	82.88	84.32	84.67	79.38	90.73	92.75	92.05	93.97
	IM3	78.25	88.46	88.14	82.29	90.15	93.45	91.20	95.39
	IM4	83.1	83.89	87.69	81.21	93.85	95.92	96.74	96.74
	IM5	87.39	82.77	84.82	86.98	91.29	90.31	93.77	91.45
	IM6	81.65	80.95	85.95	83.37	96.55	94.92	97.29	96.74
	IM7	78.24	83.69	82.83	89.65	90.61	93.64	92.57	95.83
	IM8	76.85	79.13	82.48	82.99	93.38	92.19	95.92	93.91
	IM9	86.99	81.58	88.89	81.16	91.05	91.96	91.63	93.09
	IM10	81.66	87.48	82.27	89.6	92.43	94.65	93.08	96.33

**Table 11:** Accuracy clustering by FCM, PCM, PFCM, and HPFCM, using IBSR dataset.

Image	FCM	PCM	PFCM	HPFCM
IM1	84.44	85.83	95.43	96.84
IM2	83.03	83.78	93.81	94.51
IM3	87.83	82.64	94.59	95.65
IM4	85.73	85.33	92.76	93.91
IM5	84.84	85.69	93.13	94.75
IM6	85.65	81.97	93.20	94.98
IM7	85.49	87.16	93.19	94.35
IM8	88.97	85.38	92.05	94.87
IM9	87.5	83.12	93.33	95.19
IM10	88.99	88.84	93.65	95.87
